# Vasitis: a clinical confusion diagnosis with inguinal hernia

**DOI:** 10.1590/S1677-5538.IBJU.2018.0457

**Published:** 2019-07-27

**Authors:** Chunhsuan Lin, Tsung-yi Huang

**Affiliations:** 1 Department of Urology, Kaohsiung Medical University Hospital, Kaohsiung Medical University, Kaohsiung, Taiwan

## Abstract

Vasitis or inflammation of the vas deferens is a rarely described condition categorized as either generally asymptomatic vasitis nodosa or the acutely painful infectious vasitis. Vasitis nodosa, the commonly described inflammation of the vas deferens, is benign and usually associated with a history of vasectomy. Clinically, patients present with a nodular mass and are often asymptomatic and require no specific treatment.

## CASE DESCRITION

Vasitis or inflammation of the vas deferens is a rarely described condition categorized as either generally asymptomatic vasitis nodosa or the acutely painful infectious vasitis. Vasitis nodosa, the commonly described inflammation of the vas deferens, is benign and usually associated with a history of vasectomy. Clinically, patients present with a nodular mass and are often asymptomatic and require no specific treatment (1). Infectious vasitis, thought to be caused by common urinary tract infection, is rarely reported in the literature with medical imaging readily available (2).

**Figure 1:**
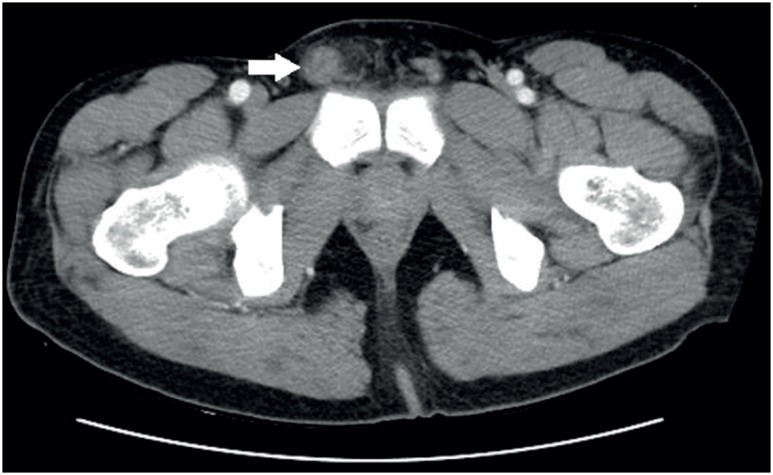
Axial computed tomography showing edematous changes and peripheral fat stranding on right spermatic cord.

**Figure 2:**
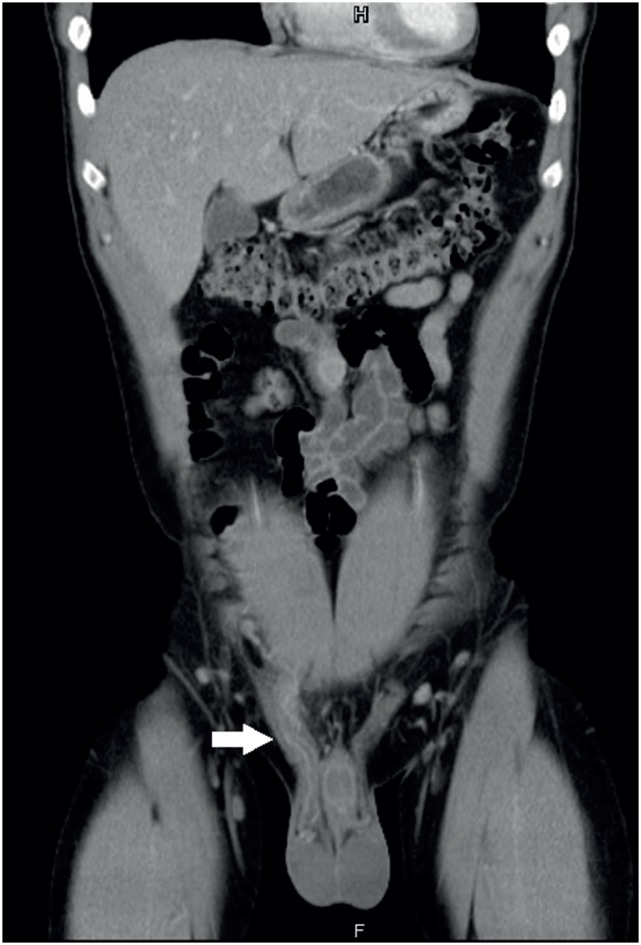
Prominent thickening of the right spermatic cord as compared to normal left spermatic cord on coronal computed tomography.

A 39-year-old man with right inguinal hernia post operation during infancy presented with right groin and scrotum tenderness for two days. He denied fever, urinary tract symptoms or previous sexually transmitted infections. Physical examination revealed a tender and palpable inguinal mass extending from the external inguinal region to the scrotum. Laboratory examination revealed leukocytosis (11.63×1000/uL) and elevated C-reactive protein level (13.09 x mg/L). Urinalysis revealed sediments of white blood cell (25-50/HPF). Bedside sonography revealed normal and symmetrical testicular sizes and blood flow. Under the suspicion of incarcerated inguinal hernia, computed tomography (CT) was arranged, which revealed prominent thickening of the right spermatic cord and vas deference with edematous changes and peripheral fat stranding (Figures 1 and 2). Empirical antibiotic treatment (ciprofloxacin) was initiated with a favorable course. On follow-up 3 months after discharge, he remained asymptomatic and pain-free.

Infectious vasitis, while rarely reported in the literature, can be difficult to distinguish from incarcerated inguinal hernia in clinical and ultrasonography findings, as both present with groin masses and pain. Laboratory results were usually normal or a slightly elevated white blood count. A few cells might be present in urine, but urine cultures are usually negative. In the few infectious vasitis cases described, imaging was not used and the patients were treated surgically for suspected inguinal hernias, but no hernia was found (3). In the present case, CT was used to differentiate between inguinal hernia and vasitis. Therefore, infectious vasitis mimicks the incarcerated inguinal hernia on clinical and sonographic examinations, but CT can readily distinguish the two diseases; thus, unnecessary surgeries can be avoided (4). Owing to chlamydia or gonorrhea, the most likely causative agent, treatment usually consists of antibiotics such as quinolones (5).
